# Ruptured Pulmonary Hydatid Cyst Presenting With Hemoptysis in a Term Pregnancy: A Case Report and Literature Review

**DOI:** 10.7759/cureus.112697

**Published:** 2026-07-15

**Authors:** Ilyass Laaribi, Samia Berrichi, Ikram Zaid, Safaa Kachmar, Houssam Bkiyar

**Affiliations:** 1 Intensive Care Unit, Mohammed VI International University Hospital, Faculty of Medicine and Pharmacy of Oujda, Mohammed First University, Oujda, MAR

**Keywords:** case report, hemoptysis, hydatid cyst, lobectomy, pregnancy, pulmonary echinococcosis

## Abstract

Hydatidosis, or cystic echinococcosis, is an anthropozoonosis caused by the larval form of *Echinococcus granulosus*. A ruptured pulmonary hydatid cyst occurring during pregnancy is rare and confronts the clinician with a dual maternal and fetal imperative. We report the case of a 31-year-old patient from a rural background, gravida 3, in the third trimester of a term pregnancy, admitted for hemoptysis following lower thoracic pain and a dry cough that had been present for two weeks. On examination, the patient was hemodynamically and respiratorily stable. Chest CT angiography revealed a cavitary lesion of the left lower lobe communicating with the bronchi, consistent with a ruptured hydatid cyst, associated with alveolar hemorrhage and without pulmonary embolism. As the pregnancy was already at term, a primary cesarean section was performed to avoid the expulsive efforts of a vaginal delivery, which were liable to aggravate the hemoptysis through the rise in intrathoracic pressure, and to secure the subsequent thoracic surgery; it resulted in the birth of a healthy newborn. A left lower lobectomy was performed two weeks later, and the maternal postoperative course was uneventful. This case illustrates the effectiveness of a sequential strategy involving a cesarean section followed by delayed surgical management in the treatment of a ruptured pulmonary hydatid cyst in a woman at term, with a favorable prognosis for both mother and child, provided that a multidisciplinary approach is adopted.

## Introduction

Hydatidosis, or cystic echinococcosis, is a parasitic disease resulting from the development in humans of the larval form of the cestode* Echinococcus granulosus* [1,2]. Humans, accidental intermediate hosts, become infected by ingesting eggs shed by the dog, the definitive host, which accounts for the predominance of the disease in rural and livestock-raising areas [1]. The disease is endemic in Morocco, as well as in the Mediterranean basin, the Middle East, South America, and Africa, where it constitutes a public health concern [2-4].

These cysts most commonly occur in the liver and the lungs, accounting for approximately 70% and 20-30% of cases, respectively. Pulmonary involvement, the second most frequent location, is often revealed by its complications, particularly the rupture of the cyst into the bronchi, with hemoptysis and hydatic vomica being the main manifestations; this rupture additionally entails a risk of bronchial flooding, secondary infection, anaphylactic reaction, and secondary dissemination [1,5,6,7,8].

The occurrence of a pulmonary hydatid cyst during pregnancy is rare, and its rupture is even more so; its exact incidence is not well established, and pulmonary localization in this setting remains exceptional, having been reported only as isolated cases and small series [9,10,11,12]. It poses specific difficulties: the limitation of radiation-based investigations, the restriction of medical options owing to the potential embryotoxicity of benzimidazoles, and the need to coordinate the surgical procedure with the course of the pregnancy [3,13]. We report a case of a ruptured pulmonary hydatid cyst revealed by hemoptysis in a pregnant woman at term. Given the advanced gestational age, the acute nature of the complication, and the constraints on both imaging and pharmacological treatment, we adopted a sequential strategy prioritizing control of the acute respiratory complication and coordinated delivery before definitive surgical management of the cyst in order to optimize both maternal and fetal safety. We describe this management and discuss its particularities in light of the literature. 

## Case presentation

The patient was a 31-year-old woman living in a rural area, gravida 3 para 2, with two living children aged 10 and 5 years, respectively, from well-monitored, full-term vaginal deliveries with good neonatal adaptation. The current pregnancy, estimated to be at term based on an imprecise last menstrual period, had been unremarkable until then.

The history of the present illness dated back to two weeks before admission, marked by lower thoracic pain associated with a dry cough. On the day of admission, the course was marked by the onset of hemoptysis, prompting the consultation.

On admission, the patient was conscious, with a Glasgow Coma Scale score of 15/15 [14] and equal, reactive pupils. She was hemodynamically stable, with a blood pressure of 130/70 mmHg and a heart rate of 87 beats per minute. From a respiratory standpoint, the pulse oxygen saturation was 99% on room air, and the respiratory rate was 18 breaths per minute, with no crackles on auscultation. The Homans sign was negative. On abdominal examination, the abdomen was soft and non-tender, with no guarding and no hepatomegaly; the uterus was consistent with a term pregnancy. Active fetal movements were present, and fetal heart sounds were clearly audible. On vaginal examination, the cervix was long, posterior, and closed, with intact membranes, no vaginal bleeding, and no uterine contractions.

Obstetric ultrasound confirmed a singleton viable intrauterine pregnancy at term, with cephalic presentation, adequate amniotic fluid volume, an intact amniotic sac, and an estimated fetal weight of 3,000 g.

Laboratory findings on admission are summarized in Table 1. The complete blood count showed no significant abnormalities, with a normal white cell count and differential and no hypereosinophilia; there was mild anemia (hemoglobin 11.8 g/dL). The coagulation workup, serum electrolytes, renal function, and liver workup were unremarkable. C-reactive protein was mildly elevated. Cardiac markers (high-sensitivity troponin and NT-proBNP) were normal, and D-dimers were elevated at 2.01 µg/mL, an elevation expected in the context of pregnancy and active bleeding and therefore nonspecific.

**Table 1:** Laboratory findings on admission. INR: international normalized ratio; aPTT: activated partial thromboplastin time; AST: aspartate aminotransferase; ALT: alanine aminotransferase; NT-proBNP: N-terminal pro-B-type natriuretic peptide.

Parameter	Result	Reference range
Complete blood count		
White blood cells	8,210	4,000-10,000 /mm³
Neutrophils	6,820	2,000-7,000 /mm³
Lymphocytes	1,080	1,000-4,000 /mm³
Eosinophils	50	< 500 /mm³
Hemoglobin	11.8	12-16 g/dL
Hematocrit	35.7	36-46 %
Platelets	245,000	150,000-400,000 /mm³
Coagulation		
Prothrombin time (activity)	100	70-100 %
INR	1.0	0.8-1.2
aPTT ratio	1.1	0.8-1.2
D-dimers	2.01	< 0.5 µg/mL
Biochemistry		
Sodium	137	135-145 mmol/L
Potassium	4.1	3.5-5.1 mmol/L
Urea	0.25	0.15-0.45 g/L
Creatinine	7	6-11 mg/L
AST	34	< 35 U/L
ALT	30	< 35 U/L
Total bilirubin	10	3-12 mg/L
C-reactive protein	6.8	< 5 mg/L
Cardiac markers		
High-sensitivity troponin	2	< 14 ng/L
NT-proBNP	50	< 125 pg/mL

**Figure 1:**
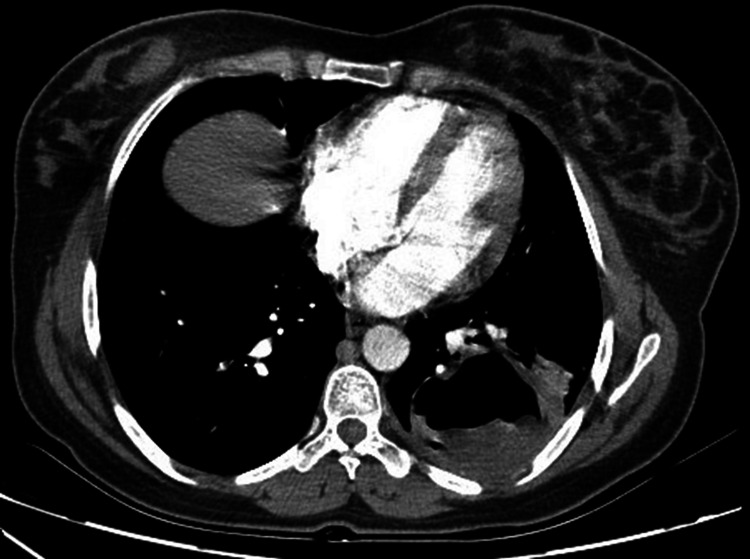
Axial chest CT image in lung window showing a cavitary lesion of the left lower lobe communicating with the bronchial tree, associated with perilesional ground-glass opacities consistent with alveolar hemorrhage, suggestive of a ruptured pulmonary hydatid cyst.

**Figure 2:**
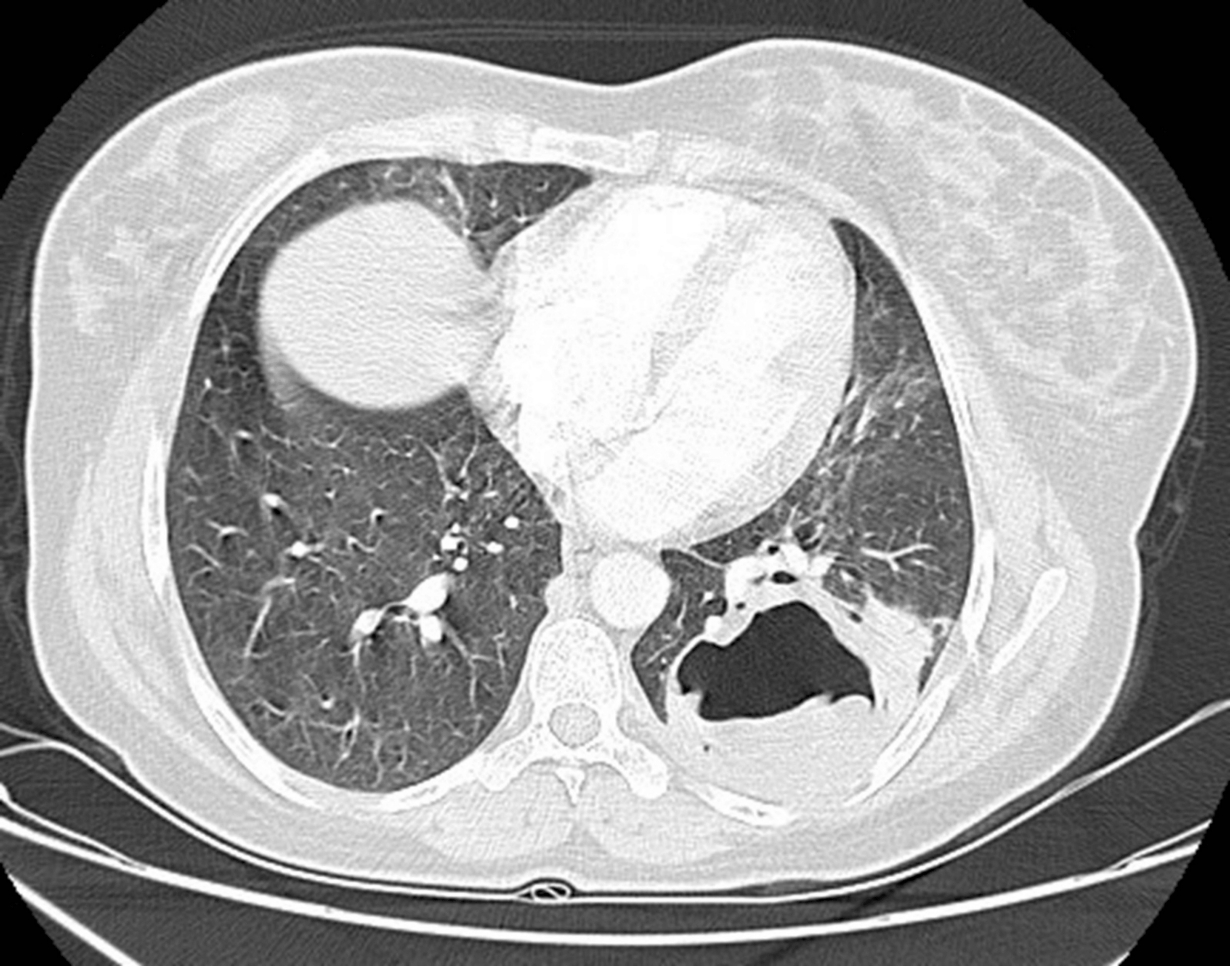
Axial contrast-enhanced chest CT image in mediastinal window showing a cavitary lesion of the left lower lobe communicating with the bronchial tree, strongly suggestive of a ruptured pulmonary hydatid cyst, with no sign of proximal pulmonary embolism.

**Figure 3:**
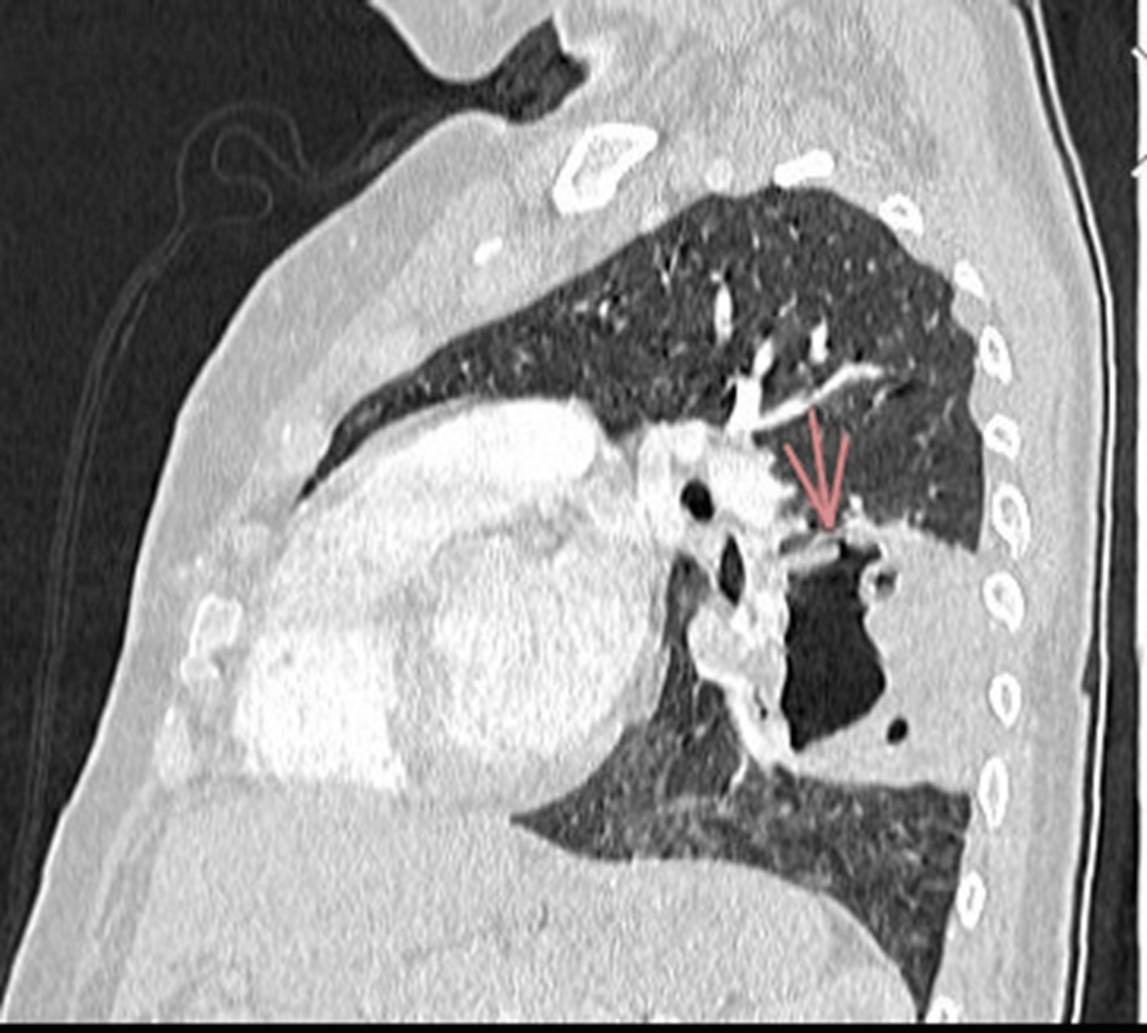
Sagittal chest CT reconstruction in lung window showing the communication between the cavity of the ruptured hydatid cyst and the bronchial tree (arrow), confirming the bronchial fistulization.

Chest CT angiography demonstrated a cavitary lesion of the left lower lobe communicating with the bronchial tree, suggestive of a ruptured pulmonary hydatid cyst, associated with perilesional ground-glass opacities consistent with alveolar hemorrhage, with no sign of pulmonary embolism (Figures 1-3). The diagnosis of a pulmonary hydatid cyst that ruptured into the bronchi, complicated by hemoptysis and alveolar hemorrhage, was thus established in a patient at term.

Prior to management, the patient received supportive treatment consisting of oxygen therapy, intravenous tranexamic acid, and close clinical monitoring. No blood transfusion or other specific interventions were required, as she remained hemodynamically and respiratorily stable. Management was then decided upon in a multidisciplinary manner, involving obstetricians, thoracic surgeons, and anesthesiologist-intensivists. As the pregnancy was already at term, continuing it until a vaginal delivery offered no additional fetal benefit. Moreover, the expulsive efforts of labor could increase intrathoracic pressure and risk aggravating the hemoptysis and promoting renewed bronchial flooding. A primary cesarean section was therefore performed to avoid these expulsive efforts and to secure the subsequent thoracic surgery by removing the constraints related to pregnancy. It resulted in the birth of a healthy male newborn, with Apgar scores of 10 and 10 at 1 and 5 minutes, respectively, and a birth weight of 3,100 g [15].

The pulmonary surgical procedure was performed two weeks after delivery through a left posterolateral thoracotomy. Intraoperatively, a ruptured pulmonary hydatid cyst with extensive destruction of the left lower lobe was identified, making a left lower lobectomy necessary. Histopathological examination of the surgical specimen confirmed the diagnosis of pulmonary hydatid disease. Albendazole was not administered during the patient's management. The postoperative course was uneventful, with removal of the chest drain on postoperative day 3 and hospital discharge on postoperative day 7. The patient subsequently underwent regular follow-up with the thoracic surgery and obstetrics teams, and the newborn was regularly followed by the pediatric team, with a favorable outcome for both mother and child to date.

## Discussion

Cystic echinococcosis remains endemic in Morocco and in many regions of the world. Close contact with animals such as dogs and sheep explains the marked predominance of the disease in rural settings, as was the case with our patient [3]. After the liver, the lung constitutes the second most frequent location, with pulmonary cysts often located in the lower lobes [1,5,6].

The natural course of a pulmonary hydatid cyst is dominated by the risk of complications, foremost among which is rupture, either bronchial or pleural. Rupture into the bronchial tree typically presents as a hydatic vomica, pathognomonic but inconstant, and as hemoptysis, as in our case; the associated alveolar hemorrhage reflects parenchymal involvement. Rupture may also be complicated by pneumothorax, pyothorax, or pleural effusion, and entails a risk of anaphylactic shock and secondary dissemination, justifying prompt management [5,9,10]. This diversity of presentations explains why a ruptured pulmonary hydatid cyst is frequently misdiagnosed and confused with pneumonia, tuberculosis, an abscess, a tumor, or a pneumothorax, particularly in pregnant women, in whom the symptoms may be wrongly attributed to the pregnancy [10].

The state of immunomodulation specific to pregnancy, in particular the decrease in cell-mediated immunity, together with the rise in placental steroids, may favor cyst growth; in the third trimester, the increase in intra-abdominal and intrathoracic pressure may further precipitate rupture, which accounts for the late presentation observed in our patient [10,13].

The diagnosis rests on a combination of epidemiological, clinical, radiological, and biological arguments. Hydatid serology lacks sensitivity in isolated pulmonary involvement, being positive in only about 50-60% of cases; hypereosinophilia is inconstant, often absent outside episodes of cyst fissuring; its absence in our patient, despite a confirmed rupture, illustrates this lack of sensitivity and should not lead to the diagnosis being ruled out [1,5,16]. As a result, imaging occupies a central role in diagnosis. In pregnant women, the use of CT must be weighed against the concern to limit fetal irradiation; nevertheless, in the third trimester, with organogenesis complete and the dose delivered during a chest examination with abdominal shielding remaining low, its benefit-risk ratio is favorable when the maternal vital prognosis is at stake, as in the context of hemoptysis [9].

The management of a ruptured pulmonary hydatid cyst is primarily surgical. Conservative techniques (cystectomy, pericystectomy, capitonnage with closure of the bronchial fistulas) are preferred whenever possible in order to preserve the parenchyma [17]. Anatomical resections, such as the lobectomy performed in our patient, are reserved for complicated cysts with substantial parenchymal destruction or when conservative surgery is not feasible; in our patient, the involvement of the left lower lobe complicated by alveolar hemorrhage prompted this choice [9,17]. Medical treatment with albendazole is usually combined with surgery to reduce the risk of recurrence, but it is contraindicated in the first trimester owing to its embryotoxicity and teratogenicity demonstrated in animals, and its safety is not established in the second and third trimesters; it is therefore usually deferred to the postpartum period [3,9,13]. In our patient, complete excision of the cyst by lobectomy and the absence of any detectable residual hydatid location led to the decision to not start albendazole, subject to clinical, serological, and radiological monitoring.

The particularity of our case lies in the coincidence of a ruptured pulmonary hydatid cyst and a term pregnancy, a situation rarely reported. The literature illustrates several strategies. Ahmed et al. described a pneumothorax revealing a ruptured and secondarily infected pulmonary hydatid cyst in a pregnant woman, treated by chest drainage followed by lobectomy via thoracotomy, with albendazole introduced after delivery, and a favorable outcome for the mother and child [9]. Menon reported a ruptured pulmonary cyst presenting as a pleural effusion in the third trimester, managed with drainage and albendazole, followed by a vaginal delivery [10]. These two cases frame the therapeutic spectrum, from simple drainage to anatomical resection, and underscore that the choice depends on the extent of the parenchymal lesions and on whether the cyst is hemorrhagic or secondarily infected.

When the pregnancy is close to term, as in our case, primary fetal delivery by cesarean section offers several advantages: it reduces the risk to the child, removes the constraints imposed by the pregnancy, and allows thoracic surgery to be performed under optimal conditions without the challenges associated with the gravid patient or anesthesia during pregnancy. Two considerations were decisive in our patient. On the one hand, as the pregnancy was already at term, undergoing vaginal delivery offered no fetal benefit, since weight and maturity had been reached. On the other hand, the expulsive efforts inherent to labor and vaginal delivery would transiently and repeatedly increase intrathoracic pressure, which, in an already ruptured and hemorrhagic cyst, would pose a risk of aggravating the hemoptysis and causing bronchial flooding; indeed, an increase in intrathoracic pressure is precisely one of the recognized mechanisms of rupture of pulmonary hydatid cysts. The cesarean section was therefore decided upon to avoid these expulsive efforts and to secure the subsequent lobectomy, rather than for an obstetric indication in itself. Performing the resection in a delayed fashion, two weeks after delivery, allowed the procedure to be carried out under safe conditions, with an uneventful course.

In the Moroccan context, Rachad et al. reported an intraperitoneal rupture of a hydatid cyst during pregnancy, recalling that the second trimester is considered the most favorable period for elective surgery in pregnant women owing to a lower risk of miscarriage than in the first trimester and of preterm delivery than in the third, but that complicated forms require emergency intervention regardless of gestational age [3]. Our case, due to the cyst's thoracic location and the term pregnancy, followed a different rationale, in which the impending delivery made it possible for the patient to undergo childbirth and the thoracic procedure at different times.

This sequential approach stabilization, delivery by cesarean section, then delayed surgical management illustrates the importance of close multidisciplinary collaboration between obstetricians, thoracic surgeons, anesthesiologists, and neonatologists. Under these circumstances, the prognosis is generally favorable, as evidenced by the uneventful course observed in the mother and the excellent condition of the child at birth.

Prevention remains a major challenge in endemic areas: deworming of dogs, control of slaughtering and veterinary inspection, hand hygiene, and health education of rural populations constitute the pillars of the fight against this preventable anthropozoonosis [3]. Prolonged clinical, serological, and radiological surveillance is recommended in order to detect in situ recurrence or a secondary location [3,18].

## Conclusions

A ruptured pulmonary hydatid cyst in a pregnant woman is a rare entity that confronts the clinician with a dual maternal and fetal imperative. Our case illustrates the effectiveness of a sequential strategy based on a primary cesarean section intended to secure a delayed lobectomy, conducted within the framework of multidisciplinary management and resulting in a favorable prognosis for both mother and child. In endemic regions such as Morocco, hydatidosis should be considered in any case of hemoptysis in a pregnant woman, and prevention remains the most effective means of reducing the burden of this parasitic disease.
